# Effect of preoperative intranasal dexmedetomidine versus placebo on intraoperative shivering in parturients undergoing cesarean section: a randomized controlled trial

**DOI:** 10.3389/fphar.2025.1661683

**Published:** 2025-11-25

**Authors:** Guolin Xu, Zhuhong Tian, Yong Ding, Jingjing Ma, Rui Li

**Affiliations:** Department of Anesthesiology and Perioperative Medicine, Second Affiliated Hospital of Anhui Medical University, Hefei, China

**Keywords:** cesarean section, epidural anesthesia, shivering, dexmedetomidine, intranasal administration

## Abstract

**Background:**

Shivering during cesarean section under epidural anesthesia is common and may negatively affect maternal comfort, surgical conditions, and physiological stability. This single-blinded, randomized controlled trial evaluated whether preoperative intranasal dexmedetomidine reduces the incidence of shivering in parturients undergoing cesarean delivery.

**Methods:**

A total of 170 parturients scheduled for elective cesarean section under continuous epidural anesthesia were randomized into a dexmedetomidine group (group DEX) or control group (group CON). The group DEX received 1 μg/kg intranasal dexmedetomidine (diluted to 1 mL), and the group CON received 1 mL intranasal normal saline approximately 30 min before anesthesia. The primary outcomes were the incidence, severity, frequency, and duration of shivering. Neonatal safety was evaluated using Apgar scores at 1 and 5 min after delivery.

**Results:**

A total of 160 patients completed the study. The incidence, frequency, severity, and duration of intraoperative shivering reactions were significantly lower in the group DEX than in the group CON (*P* < 0.01). Compared with those in the group CON, there were significantly more patients with level 2 sedation in the group DEX (*P* < 0.01). There were no significant differences in maternal hypotension, bradycardia, or nausea and vomiting events between the two groups (P > 0.01). There were no significant differences in Apgar scores between the two groups (*P* > 0.05).

**Conclusion:**

Preoperative intranasal administration of dexmedetomidine effectively prevents intraoperative shivering in parturients undergoing cesarean section.

**Clinical Trial Registration:**

https://www.chictr.org.cn identifier: ChiCTR2400079811.

## Introduction

Neuraxial anesthesia, commonly used in cesarean sections, has the advantage of minimal impact on both the mother and the fetus (Feng et al., 2021). However, up to 53% of patients experience shivering as a common adverse effect (Lamontagne et al., 2019). Shivering is an involuntary, oscillatory muscle activity that increases metabolic thermogenesis. It can lead to several harmful effects, including increased maternal oxygen consumption, higher intraocular and intracranial pressure, interference with vital sign monitoring, and exacerbation of postoperative pain (Wodarski et al., 2020). Hence, the absence of shivering can lead to a more positive surgeon’s experience.

Non-pharmacological measures such as active skin surface warming, warmed intravenous fluids, and maintaining ambient temperature are effective but may be limited by practical constraints in the obstetric setting (Qi et al., 2022). Pharmacological interventions can modulate central thermoregulatory control mechanisms, offering a potential solution for reducing perioperative shivering. Previous studies have shown that opioids, 5-HT_3_ receptor antagonists, and α_2_-adrenoceptor agonists can reduce the occurrence of maternal intraoperative shivering (Liu et al., 2018).

Dexmedetomidine, an α_2_-adrenergic receptor agonist, administered intravenously, can reduce shivering in parturients during surgery (Lamontagne et al., 2019; Sween et al., 2021). Recent research suggests that dexmedetomidine is rather rapidly and efficiently absorbed after intranasal administration. Compared with intravenous administration, intranasal administration may be a feasible alternative (Kuang et al., 2022). However, the effectiveness of intranasal dexmedetomidine in preventing maternal intraoperative shivering remains unclear.

This single-blinded, randomized controlled trial was designed to test the hypothesis that intranasal dexmedetomidine can prevent shivering reactions in parturients undergoing cesarean section.

## Methods

### Ethics

Ethical approval for this study (Approval No. YX2023-144 [F1]) was granted by the Ethics Committee of the Second Affiliated Hospital of Anhui Medical University, Hefei, China (Chairperson: Prof. Y. Zhang) on 9 November 2023. The study protocol was registered in the Chinese Clinical Trial Registry (https://www.chictr.org.cn, ChiCTR2400079811), and the trial adhered to the CONSORT guidelines.

### Design

This is a single-center, single-blinded, randomized controlled trial.

### Eligibility and randomization

A total of 170 parturients scheduled for elective cesarean section under epidural anesthesia between 15 January 2024, and 1 September 2024, were enrolled. Inclusion criteria were age >18 years and ASA physical status II–III. In obstetric patients, ASA II includes healthy women or those with mild systemic disease, whereas ASA III includes women with well-controlled systemic conditions such as mild hypertension or hypothyroidism. Normal physiological changes of pregnancy were considered when assigning ASA classification.

Exclusion criteria included: emergency cesarean delivery, general anesthesia, body weight <60 kg or >120 kg, known allergy to dexmedetomidine, severe cardiac, renal, or hepatic dysfunction, preeclampsia or eclampsia, combined spinal–epidural anesthesia, and intraoperative blood transfusion.

Randomization was performed using a computer-generated random number table. Eligible participants were assigned to either the group DEX or the group CON. Group allocations were concealed in sequentially numbered, sealed, opaque envelopes prepared by an independent investigator not involved in patient management. Upon enrollment, the attending anesthesiologist opened the next envelope to determine group allocation. The study followed a single-blind design, patients were blinded to group assignment, whereas the anesthesiologist administering the study drug and performing the epidural block was aware.

### Interventions

Upon entering the operating room, patients underwent continuous monitoring of their electrocardiogram, pulse oximetry, and noninvasive blood pressure. An electronic thermometer was used to measure the oropharyngeal temperature before and during surgery.

For epidural catheter insertion, the L2-L3 interspace was selected, and the catheter was advanced 4 cm into the epidural space. To confirm the correct placement, 3 mL of 2% lidocaine solution was injected through the catheter. Subsequently, 10–20 mL of 0.75% ropivacaine was administered incrementally to establish epidural anesthesia while avoiding excessively high anesthesia levels.

Patients in the group DEX received an intranasal dose of 1 μg/kg dexmedetomidine, which was diluted with 0.9% saline to a final volume of 1 mL. This solution was carefully administered intranasally, drop by drop, using a needleless 1 mL syringe, approximately 30 min before epidural anesthesia (Iirola et al., 2011). In the group CON, patients received 1 mL of 0.9% saline intranasally using the same method, ensuring consistency between the two groups. The operating theatre ambient temperature was maintained at 23–25 °C throughout the procedures, with no active warming measures applied. During the perioperative period, all patients were provided with supplemental oxygen via a mask at a flow rate of 4 L/min.

### Primary outcome

The primary outcome was the evaluation of the effect of intranasal dexmedetomidine instillation on maternal intraoperative shivering reactions, including the incidence, frequency, duration, and severity of shivering.

The severity of shivering was assessed using a five-point grading scale: Grade 0: No shivering. Grade 1: Presence of one or more of the following—piloerection, peripheral vasoconstriction, or peripheral cyanosis—without visible muscle activity. Grade 2: Visible muscle activity confined to one muscle group. Grade 3: Visible muscle activity involving more than one muscle group. Grade 4: Gross muscle activity involving the whole body. For patients who developed grade 3 or 4 shivering after epidural anesthesia, a forced-air warming blanket was used as a remedial measure (Lamontagne et al., 2019).

### Secondary outcome

The secondary outcomes included nausea, vomiting, sedation levels, blood pressure, and heart rate, which were recorded to evaluate the effects of intranasal dexmedetomidine in patients undergoing cesarean section. Sedation levels were assessed using a four-point scale: Grade 1: Awake and alert. Grade 2: Drowsy but responsive to verbal stimuli. Grade 3: Drowsy, arousable to physical stimuli. Grade 4: Unarousable.

Interventions for hemodynamic changes included administering intravenous atropine (0.5 mg) if the heart rate fell below 60 bpm and intravenous phenylephrine (40 μg) if blood pressure dropped below 20% of the baseline value. The oropharyngeal temperature was measured preoperatively and intraoperatively using an electronic thermometer. At the end of the surgery, satisfaction with anesthesia was evaluated by both patients and surgeons using a 0–10-point satisfaction scale.

Additionally, the Apgar score was assessed 1 and 5 min after delivery to determine the effect of intranasal dexmedetomidine on neonatal outcomes. Other recorded outcomes included general patient information, operative time, total ropivacaine dosage, total fluid input, blood loss, and perioperative biochemical indices (Lamontagne et al., 2019).

### Sample size

Based on previous studies of intravenous dexmedetomidine for the prevention of shivering, the estimated difference in mean shivering scores between groups was 0.58, with a standard deviation of 1.05. Using a two-sided α = 0.05 and β = 0.10 (power = 90%), the required sample size was calculated to be 70 patients per group. Allowing for a 20% dropout rate, the total planned enrollment was adjusted to 168 patients. Therefore, 170 parturients were recruited and randomized into the group DEX and group (Nesioonpour et al., 2022).

### Statistical analyses

Normally distributed data were presented as means ± standard deviations, non-normally distributed data as medians (interquartile ranges), and categorical data as frequencies or percentages. A t-test was used to compare normally distributed continuous variables, whereas a nonparametric test was used for non-normally distributed data. Categorical variables were analyzed using the chi-square test. A *P* value of less than 0.05 was considered statistically significant for all tests.

## Results

**FIGURE 1:**
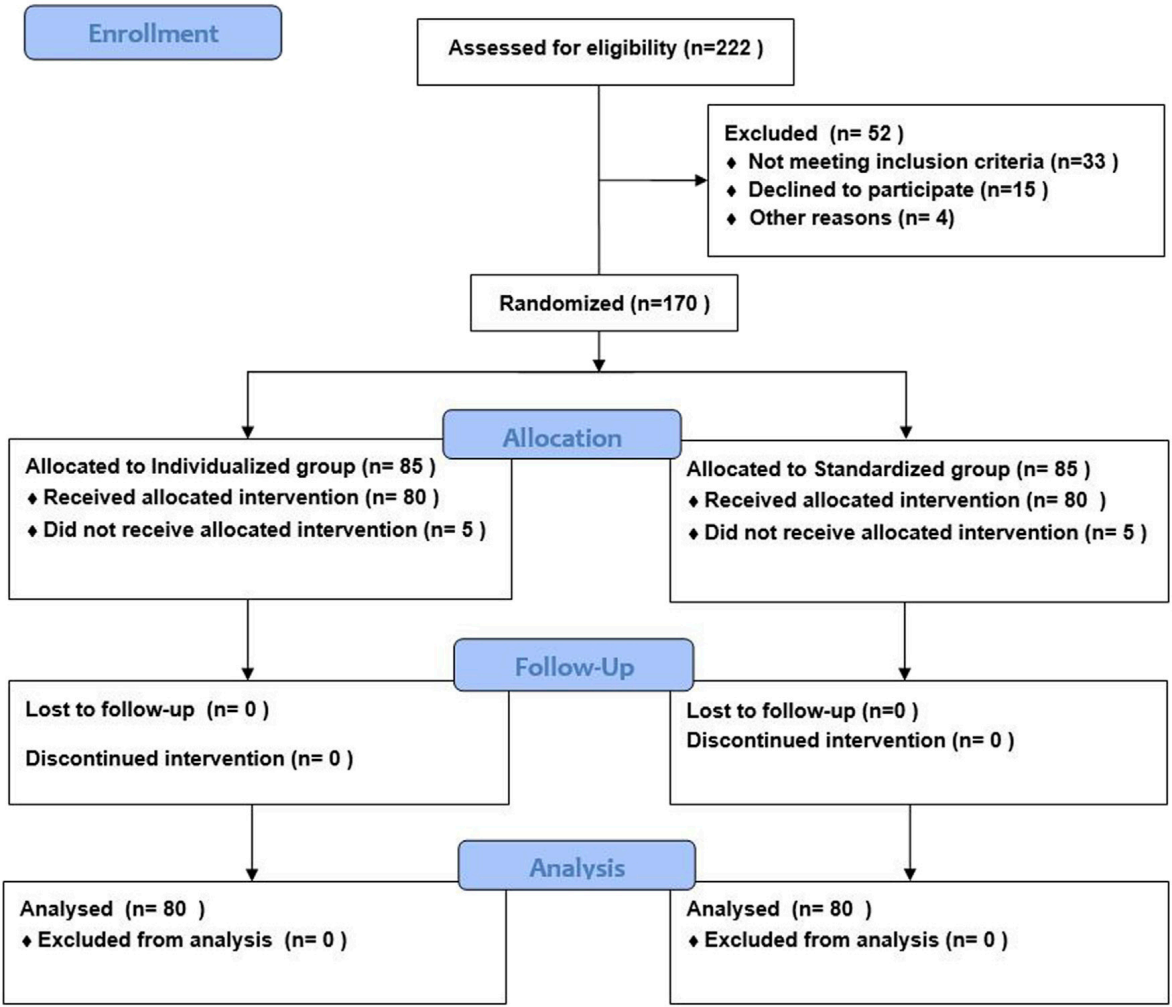
Flowchart illustrating the study's participant allocation process. Initially, 222 participants were assessed for eligibility; 52 were excluded. There maining 170 were randomized into two groups: 85 in the group CON and 85 in the group DEX. In both groups, 80 participants received the intervention, while five did not. No participants were lost to followup or discontinued. Analysis included all 80 participants in each group.

A total of 170 patients were enrolled between 15 January 2024, and 1 September 2024, and randomly divided into two groups; five patients in each group were excluded due to changes in anesthesia methods, resulting in 160 patients completing the study (Figure 1).

### Baseline characteristics

There were no significant differences between the two groups regarding age, height, weight, gestational age, ASA classification, complications, parity, or room temperature (*P* > 0.05). Similarly, no statistically significant differences were observed in the preoperative body temperature, mean arterial pressure, heart rate, or SpO_2_ between the groups (*P* > 0.05) (Table 1). Notably, no opioids or 5-HT3 receptor antagonists were administered intraoperatively in this study.

**TABLE 1:** Sociodemographic and surgical characteristics.

Parameters	CON (n = 80)	DEX (n = 80)	*P*-value
Age (year)	31.0 (28.0, 33.0)	32.0 (29.0, 35.0)	0.080^a^
Weight (kg)	70.0 (65.0, 77.0)	71.0 (65.0, 78.0)	0.425^a^
BMI (kg/m^2)^	27.5 ± 2.8	28.0 ± 3.2	0.333^b^
ASA			0.428^c^
Ⅱ	45 (56.2%)	40 (50.0%)	
Ⅲ	35 (42.8%)	40 (50.0%)	
Gestational age (weeks)	39.1 (38.5, 39.6)	39.0 (38.2, 39.4)	0.062^c^
Complication			
Diabetes	16 (20%)	20 (25%)	0.450^c^
Hypothyroidism	10 (8.0%)	15 (18.8%)	0.270^c^
Hypertension	1 (1.3%)	3 (3.8%)	0.310^c^
Primipara			0.426^c^
NO	42 (52.5%)	47 (58.8%)	
YES	38 (47.5%)	33 (41.3%)	
Preoperative body temperature (°C)	36.6 (36.5, 36.7)	36.6 (36.4, 36.8)	0.775^a^
Preoperative MAP (mmHg)	87.7 (83.2, 92.9)	89.0 (81.2, 94.7)	0.563^a^
Preoperative heart rate (bpm)	87.5 (80.3, 99.5)	87.5 (83.0, 96.8)	0.804^a^
Preoperative SpO2 (%)	98.0 (97.0, 99.0)	98.0 (98.0, 99.0)	0.709^a^
Room temperature (°C)	25.1 (24.9, 25.4)	25.1 (25.0, 25.3)	0.770^a^

Data are presented as median (Quartile), numbers (proportions).

^a^ Kruskal–Wallis (H) test.

^c^ chi-square test.

^b^ t-test. *P* < 0.05 is statistically significant. CON, intranasal saline; DEX, intranasal dexmedetomidine.

Abbreviations: ASA, american society of anesthesiologist; MAP, mean arterial pressure; SpO2, pulse oxygen saturation.

### Primary outcomes

Forty patients (50.0%) in the group CON and 10 patients (12.5%) in the group DEX experienced shivering reactions, with a statistically significant difference in the incidence (*P* < 0.001). The number, severity, and duration of shivering episodes were significantly lower in the group DEX than in the group CON (*P* < 0.001). However, no statistically significant differences were observed between the two groups in the time to the first shivering episode after anesthesia (Table 2).

**TABLE 2:** Incidence and severity of shivering.

Characteristics of shivering	CON (n = 80)	DEX (n = 80)	*P*-value
Shivering			<0.001^a^
NO	40 (50.0%)	70 (87.5%)	
YES	40 (50.0%)	10 (12.5%)	
Severity of shivering (%)			<0.001^a^
Grade 0	40 (50.0%)	70 (87.5%)	
Grade 1	3 (7.5%)	1 (10.0%)	
Grade 2	9 (22.5%)	3 (30.0%)	
Grade 3	20 (50.0%)	5 (50.0%)	
Grade 4	8 (20.0%)	1 (10.0%)	
Total number of shivering	0.5 (0.0, 1.0)	0.0 (0.0, 0.0)	<0.001^b^
First shivering time after anesthesia (minutes)	7.0 (6.0, 8.8)	8.0 (5.0, 24.3)	0.344^b^
Shivering duration (minutes)	0.0 (0.0, 11.0)	0.0 (0.0, 0.0)	<0.001^b^

Data are presented as median (Quartile), numbers (proportions).

^a^ chi-square test. P < 0.05 is statistically significant.

^b^ Kruskal–Wallis (H) test.

CON, intranasal saline; DEX, intranasal dexmedetomidine.

Grade 0: No shivering. Grade 1: Piloerection or peripheral vasoconstriction, but no visible shivering. Grade 2: muscular activity in only one muscle group. Grade 3: muscular activity in more than one muscle group, but not generalized. Grade 4: shivering involving the whole body. The severity of shivering is recorded as the most severe grade that occurs in the surgery.

### Sedation and hemodynamic stability

**FIGURE 2:**
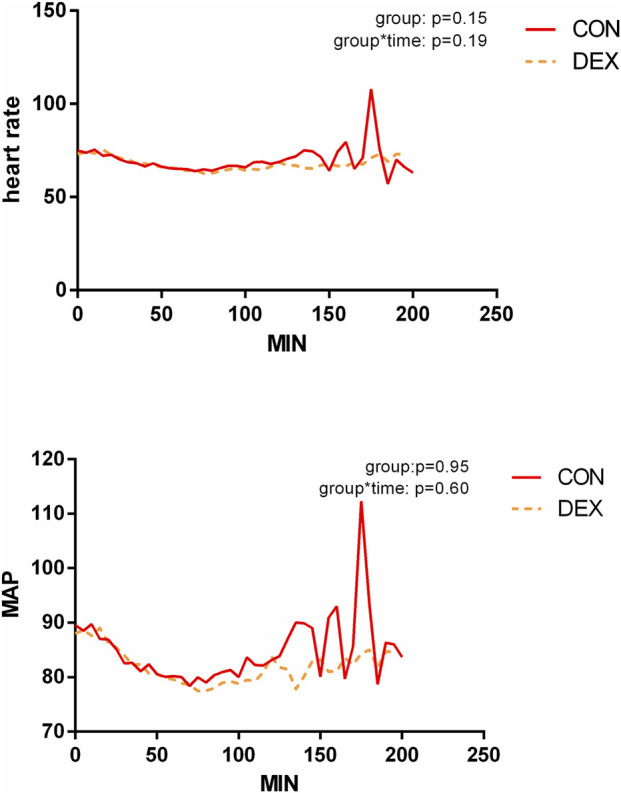
Perioperative MAP and heart rate. Two line graphs compare heart rate and mean arterial pressure (MAP) over time in two groups: CON (solid red line) and DEX (dashed orange line). The heart rate graph shows slight variations, with a spike in the group CON around 200 minutes. The MAP graph reflects similar trends with a spike in the group CON. P-values for group and group-time interactions are included, indicating no significant differences.

The maximum sedation level was Grade 3 (drowsiness), experienced by one patient (1.3%) in the group CON and 45 patients (56.3%) in the Group DEX. Blood pressure and heart rate did not differ significantly between the groups, with no differences in systolic blood pressure (SBP), diastolic blood pressure (DBP), or heart rate variability. Hypotension occurred in 21 patients (26.3%) in the group CON and 16 patients (20.0%) in the Group DEX, while bradycardia occurred in only one patient (1.3%) in the group DEX. None of the patients developed hypoxemia. Nausea and vomiting were reported in 19 patients (23.8%) in the group CON and in 14 patients (17.5%) in the group DEX, with no statistically significant difference (*P* > 0.05) (Figure 2; Table 3).

**TABLE 3:** Perioperative parameters.

Parameters	CON (n = 80)	DEX (n = 80)	*P*-value
Duration of surgery (minutes)	60.0 (51.8, 72.3)	63.5 (57.0, 72.8)	0.350^a^
Sedation level (%)			<0.001^b^
1	1 (1.3%)	0 (0.0%)	
2	78 (97.5%)	35 (43.8%)	
3	1 (1.3%)	45 (56.3%)	
4	0 (0.0%)	0 (0.0%)	
Nausea and vomiting (%)	19 (23.8%)	14 (17.5%)	0.330^b^
Minimum heart rate (bpm)	72.5 ± 9.6	70.7 ± 9.4	0.238^c^
Bradycardia (%)	0 (0.0%)	1 (1.3%)	0.320^b^
Hypotension (%)	21 (26.3%)	16 (20.0%)	0.310^b^
Coefficient of SBP variation	8.0 (6.5, 10.7)	8.6 (6.2, 11.1)	0.584^a^
Coefficient of DBP variation	13.5 (10.7, 16.8)	13.1 (10.5, 16.6)	0.810^a^
Coefficient of HR variation	10.3 (8.2, 12.5)	11.4 (9.3, 13.6)	0.019^a^
Satisfaction of patient	7.0 (6.0, 8.0)	8.5 (7.0, 9.0)	<0.001^a^
Satisfaction of surgeons	7.0 (6.0, 8.0)	9.0 (7.3, 9.0)	<0.001^a^

Data are presented as Mean ± standard deviation median (Quartile), and numbers (proportions).

^a^ Kruskal–Wallis (H) test.

^b^ chi-square test.

^c^ t-test. P < 0.05 is statistically significant.

CON, intranasal saline; DEX, intranasal dexmedetomidine. Grade 1: awake and alert, Grade 2: drowsy, responsive to verbal stimuli, Grade 3: drowsy, arousable to physical stimuli, Grade 4: unarousable.

Abbreviations: IQR, interquartile range.

### Satisfaction and neonatal outcomes

Patient and surgeon satisfaction scores were significantly higher in the group DEX than in the group CON (*P* < 0.001) (Table 3). The Apgar scores at 1 and 5 min were consistent between the two groups (median [IQR]: 10 [10, 10]), with no statistically significant differences (*P* > 0.05).

### Temperature and other outcomes

**FIGURE 3:**
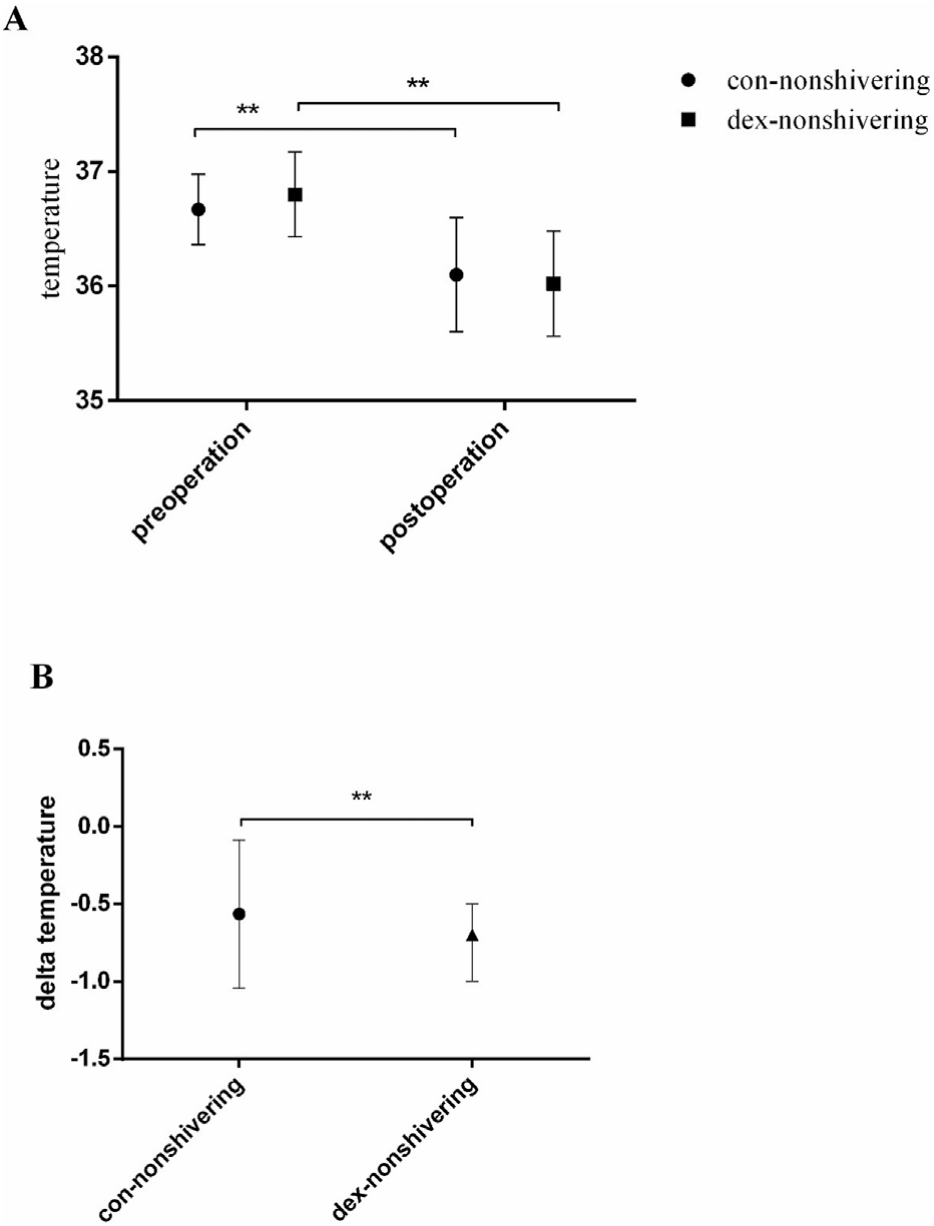
Perioperative body temperature. Graph A shows temperature. comparison between con-nonshivering and dex-nonshivering groups preoperation and postoperation, with a significant difference (** is *P* < 0.05); Graph B illustrates delta temperature differences between the two groups, also with a significant difference (** is *P* < 0.05).

Among the patients who did not develop shivering, the temperature difference was significantly greater high in the group DEX than in the group CON (*P* < 0.05) (Figure 3). No significant differences were observed in perioperative biochemical indices, ropivacaine consumption, intraoperative phenylephrine use, bleeding volume, infusion volume, or fetal delivery time between the two groups (*P* > 0.05) (Supplementary Table 1; Table 2).

## Discussion

This study demonstrated that preoperative intranasal dexmedetomidine significantly reduced the incidence, duration, and severity of shivering in parturients undergoing cesarean section under epidural anesthesia. These findings highlight that, with the exception of a mild sedative effect, intranasal dexmedetomidine had no significant adverse effects on parturients or their newborns.

Previous studies have reported that the incidence of shivering reactions in patients undergoing cesarean section under epidural anesthesia is approximately 55%, which aligns with the 50% incidence observed in the group CON of this study (Lamontagne et al., 2019; Azemati et al., 2022). In contrast, the incidence of shivering in the group DEX was significantly lower (12.5%), with a marked reduction in both the duration and severity of the shivering episodes. These results suggest that intranasal dexmedetomidine is effective in the prevention of shivering during cesarean sections under epidural anesthesia.

Substantial evidence exists regarding the use of intravenous dexmedetomidine to prevent and treat shivering reactions. For instance, Nesioonpour et al. found that intravenous dexmedetomidine could reduce shivering scores, and Lamontagne et al. reported that intravenous dexmedetomidine was effective in treating maternal shivering, with 90% of cases resolving within 10 min post-injection (Lamontagne et al., 2019; Nesioonpour et al., 2022). Similarly, Mona et al. demonstrated that intravenous dexmedetomidine could prevent postoperative shivering in patients undergoing abdominal surgery, reducing the incidence from 50% to 12.5%, which is consistent with our findings for intranasal dexmedetomidine (Mona et al., 2021).

The mechanism of shivering during epidural anesthesia is complex. Shivering is often triggered when the core body temperature decreases by 0.5 °C or reaches the shivering threshold. Under epidural anesthesia, factors such as redistribution of body heat, loss of thermoregulatory vasoconstriction, and reduced sweating threshold contribute to shivering (Tan Sook Kuan et al., 2023). Additionally, recent studies have suggested that intraoperative anxiety may exacerbate shivering (Jeevan et al., 2023). The exact mechanism by which dexmedetomidine prevents shivering is not fully understood, but it is thought to involve α2-adrenoceptor agonism in the locus coeruleus and spinal cord, both of which exert sedative and antinociceptive effects, as well as a sympatholytic effect that regulates body temperature by modulating sympathetic nervous system activity (Chang et al., 2022). Our results suggest that intranasal dexmedetomidine likely prevents shivering by regulating the body’s thermoregulatory center through these mechanisms. Still, the decrease in body temperature in the patients with dexmedetomidine but without shivering was more important than in the patients in the control group without shivering, suggesting that the effect of dexmedetomidine in controlling shivering is independent of body temperature, possibly by lowering the thresholds for shivering and vasoconstriction (Kim et al., 2025). Additional studies are necessary to elucidate those points.

Intranasal dexmedetomidine offers unique advantages over intravenous administration. Intranasal administration offers several advantages, including a slower and more stable onset of action, high safety, no liver first-pass effect, ease of use, and non-invasiveness, making it particularly suitable for clinical settings where patient comfort and compliance are important. In contrast, intravenous administration, while providing a more rapid effect, may be associated with higher risks of adverse reactions and requires venous access, which can be inconvenient in certain scenarios (He et al., 2023). Moreover, intranasal administration of dexmedetomidine may achieve effective results at low blood drug concentrations (Yu et al., 2019). In this study, a dose of 1 μg/kg intranasally was comparable to the intravenous doses used in previous studies but with a lower plasma concentration (0.25 ng/mL vs. >1 ng/mL). This lower concentration may reduce the risk of side effects commonly associated with higher blood concentrations, such as hypotension and bradycardia. While intravenous dexmedetomidine has a faster onset, intranasal administration takes 30–75 min to reach peak effect, which is why we administered it 30 min before anesthesia (Li et al., 2018).

In terms of safety, this study found no significant impact of intranasal dexmedetomidine on maternal vital signs, including blood pressure and heart rate. Although the sedation levels were higher in the group DEX, no excessive sedation or complications occurred. The sedation observed in the group DEX was mild, resembling natural sleep, and the patients could be easily aroused. Previous studies have shown that dexmedetomidine, both intravenous and intranasal, has a low incidence of adverse effects like nausea, vomiting, and severe cardiovascular events (Azemati et al., 2022). Additionally, intranasal dexmedetomidine had no significant impact on neonatal Apgar scores, which is consistent with other studies showing no adverse effects on newborns in cesarean sections (Ojha et al., 2022).

Dexmedetomidine is a pregnancy category C medication; animal studies have shown adverse effects, but there are no adequate human studies. Therefore, it can be used during pregnancy only if the potential benefits outweigh the potential risks to the fetus. Furthermore, it is already well-known that IV dexmedetomidine can be used safely in obstetric patients to induce sedation and control shivering, anxiety, postoperative nausea and vomiting, and postpartum depression. Nevertheless, it is true that data regarding fetus transfer are conflicting, as reviewed recently, but dexmedetomidine displays a high placental retention ratio (maternal/fetal index of 0.77), meaning that only a small amount transfers to the fetus. In the present study, intranasal dexmedetomidine was used to have a direct effect on the brain by bypassing the blood-brain barrier. Furthermore, dexmedetomidine was given right before epidural anesthesia, which was performed right before cesarean section. When given intranasally, peak plasma concentration is reached 38 min after administration. In the absence of complications and delays, the cesarean section can be completed when peak concentration is reached. Therefore, considering the intranasal administration and the timing of administration, the fetal exposure was probably minimal, but it will have to be confirmed (Iirola et al., 2011; Douglas et al., 2025).

Importantly, patient and surgeon satisfaction scores were higher in the group DEX, suggesting that intranasal dexmedetomidine not only improved clinical outcomes but also enhanced the overall experience of both the parturient and the surgical team. The study’s findings highlight that intranasal dexmedetomidine can effectively prevent shivering without significantly altering other perioperative indicators. This study represents a significant advancement in maternal anesthesia, as it is the first to explore the preventive use of intranasal dexmedetomidine for shivering during cesarean sections under epidural anesthesia. This approach is simpler than intravenous administration, is easier to administer, and offers similar clinical benefits. Given its ease of use and effectiveness, intranasal dexmedetomidine may be a valuable tool for optimizing obstetric anesthesia.

However, this study has several limitations. It was a single-center, single-blind trial, which may have introduced bias. Additionally, the study did not include a comparison with intravenous dexmedetomidine, which could have provided further insight into the relative effectiveness of the two routes. Plasma concentrations of dexmedetomidine were also not measured. Future multicenter studies and pharmacokinetic analyses are needed to better define optimal dosing, timing, and fetal exposure. Despite these limitations, this study demonstrated that intranasal dexmedetomidine is a promising option for preventing shivering reactions during cesarean sections.

## Conclusion

Preoperative intranasal administration of dexmedetomidine effectively prevents intraoperative shivering in parturients undergoing cesarean section, without significant adverse effects on maternal hemodynamics, sedation, or neonatal outcomes.

## Data Availability

The raw data supporting the conclusions of this article will be made available by the authors, without undue reservation.
