# Supplementary material for: Effect of preoperative intranasal dexmedetomidine versus placebo on intraoperative shivering in parturients undergoing cesarean section: a randomized controlled trial
Source: Front Pharmacol. 2025 Nov 25;16:1661683. doi: 10.3389/fphar.2025.1661683 (PMC12685831; doi:10.3389/fphar.2025.1661683)
Supplement: Supplementary file 1 [file Table1.docx]

| **Supplement table 1 Perioperative biochemical index** | | |  |
| --- | --- | --- | --- |
|  | **CON(n=80)** | **DEX(n=80)** | ***P*-value** |
| Preoperative WBC (×10⁹/L) | 8.8±2.0 | 8.8±1.2 | 0.910 ^c^ |
| Preoperative Hb (g/L) | 113.4±7.8 | 116.9±14.6 | 0.790 ^c^ |
| Postoperative WBC (×10⁹/L) | 12.5±2.1 | 11.1±1.6 | 0.260 ^c^ |
| Postoperative Hb (g/L) | 98.5±16.9 | 100.5±11.8 | 0.300 ^c^ |
| Preoperative Alt (U/L) | 10.5±3.1 | 14.9±7.9 | 0.430 ^c^ |
| Preoperative Ast (U/L) | 16.8±4.8 | 16.1±3.3 | 0.270 ^c^ |
| **Notes:** Data are presented as Mean ± standard deviation. CON = intranasal saline, DEX = intranasal dexmedetomidine.  **Abbreviations**:WBC; White Blood Cells; Hb, Hemoglobin; ALT alanine aminotransferase; AST, glutamic oxalacetic transaminase | | | |
