# Supplementary material for: Effect of preoperative intranasal dexmedetomidine versus placebo on intraoperative shivering in parturients undergoing cesarean section: a randomized controlled trial
Source: Front Pharmacol. 2025 Nov 25;16:1661683. doi: 10.3389/fphar.2025.1661683 (PMC12685831; doi:10.3389/fphar.2025.1661683)
Supplement: Supplementary file 2 [file Table2.docx]

**Supplement table 2 Intraoperative clinical characteristics**

|  | **CON (n=80)** | | **DEX(n=80)** | ***P*-value** | | |  |
| --- | --- | --- | --- | --- | --- | --- | --- |
| Total volume of ropivacaine(mL) | 13.0 (11.0,14.0) | 13.0 (11.0, 15.0) | | | 0.301 |  |  |
| Dosage of Deoxyepinephrine(µg) | 0.0 (0.0, 40.0) | 0.0 (0.0, 0.0) | | | 0.130 |  |  |
| Total intravenous fluid (mL) | 700.0 (500.0, 800.0) | 700.0 (600.0, 800.0) | | | 0.128 |  |  |
| Estimated blood loss (mL) | 400.0 (400.0, 400.0) | 400.0 (400.0, 400.0) | | | 0.395 |  |  |
| Time to extraction (minutes) | 6 (3) | 6 (4) | | | 0.920 |  |  |
| Duration in PACU(min) | 32.0 (24.0, 35.0) | 28.5 (26.0, 33.0) | | | 0.545 |  | |

Notes: Data are presented as median (IQR), numbers (proportions), Mean± standard deviation. CON = intranasal saline, DEX = intranasal dexmedetomidine.
